# Impact of age at diagnosis of type 1 diabetes on the occurrence of celiac disease in children

**DOI:** 10.5339/qmj.2026.27

**Published:** 2026-06-10

**Authors:** Dalal Aicha Chellali, Ben Mehel Benakriche, Reda Belbouab, Mustapha Noumi

**Affiliations:** Laboratory of Animal and Applied Physiology, Abdelhamid Ibn Badis University, Mostaganem, Algeria; Laboratory of Nutrition Physiology and Food Safety, Ahmed Ben Bella University, Oran, Algeria; Department of Pediatrics, Mustapha Bacha University Hospital, Algiers, Algeria

**Keywords:** Type 1 diabetes mellitus, celiac disease, gluten, children, age at diagnosis, Algeria

## Abstract

**Introduction:**

Type 1 diabetes mellitus (T1DM) and celiac disease (CD) represent two frequently recognized and interrelated autoimmune disorders. Despite the well-documented coexistence of T1DM and CD, the exact etiology behind this association remains unclear.

**Methods:**

A retrospective study analyzed handwritten medical files of 109 children and adolescents diagnosed with T1DM documented between the years 2004 and 2022 in the Pediatric Unit of Mustapha Bacha University Hospital, Algiers, Algeria. Data were collected, including age, sex, age at diagnosis of both T1DM and CD, the mean annual Glycated Hemoglobin ( HbA1c) levels, celiac serology, and histopathological results. Comparisons between the CD-negative and CD-positive groups were performed using the Mann–Whitney U test.

**Results:**

The mean age was 9.99 ± 4.05 years, and the age at diagnosis of T1DM was 6.33 ± 3.45 years. HbA1c was 8.47 ± 1.42%, equivalent to 69.1 ± 15.5 mmol/mol. Celiac serology was positive in 21 patients (19.3%), including 11 patients with a confirmed CD (57.89%), with a mean age at CD diagnosis of 5.82 ± 2.82 years. The Mann–Whitney U test revealed a significant difference in the age at diagnosis of T1DM (P = 0.027). Logistic regression analysis showed that age at T1DM diagnosis was significantly associated with the risk of developing CD, with a 21.4% decrease in risk per additional year of age (odds ratio = 0.786 [95% CI, 0.627–0.986]; P = 0.038).

**Conclusion:**

In this retrospective cohort, younger age at T1DM diagnosis was associated with a higher risk of CD, supporting intensified screening in children diagnosed at younger ages. Prospective studies are warranted to confirm these clinical implications.

## 1. INTRODUCTION

Type 1 diabetes mellitus (T1DM) is a common childhood autoimmune disease marked by β-cell destruction and resulting in absolute insulin deficiency.^1^ Algeria has one of the highest incidences of T1DM in children worldwide, ranking sixth according to the International Diabetes Federation (IDF).^2^

Celiac disease (CD) is defined as an autoimmune enteropathy triggered by gluten ingestion in genetically predisposed individuals, marked by a chronic inflammatory response and damage in the small intestinal mucosa.^3^

The prevalence of CD in patients with T1DM ranges between 1% and 10%, compared to 0.3% to 1% in the general population.^4^ In western Algeria, a serological screening of 116 newly diagnosed children with T1DM (1993–1994) reported a 16.4% to 20.0% prevalence of CD using Immunoglobuline A( IgA)/ Immunoglobuline G(IgG) antigliadin and IgA anti-endomysial antibodies.^5^

The co-occurrence of CD and T1DM in pediatric populations is associated with multiple complications, including growth faltering and nutritional disturbances, impaired glycemic control, gastrointestinal issues, and an elevated risk of additional autoimmune conditions,^6^ as well as reduced bone mineral density.^7^

Due to these severe complications, our retrospective study aimed to describe the clinical characteristics of children and adolescents with T1DM and to analyze the influence of age at T1DM diagnosis on CD onset in Algerian children and adolescents with T1DM.

## 2. PATIENTS AND METHODS

### 2.1 Study design

This was a retrospective study involving a review of handwritten medical records of children and adolescents with T1DM, with or without CD, recorded between 2004 and 2022, in the pediatric unit of Mustapha Bacha University Hospital in Algiers, Algeria, one of the largest hospitals in the country. The hospital primarily serves an urban population but also receives patients from various regions across the country.

### 2.2 Study population

The study population included 109 patients with an age between 2 and 18 years with confirmed diagnoses of T1DM and CD as per the respective guidelines of the International Society for Pediatric and Adolescent Diabetes (ISPAD)^8^ and the European Society for Paediatric Gastroenterology Hepatology and Nutrition (ESPGHAN).^9^ Patients with type 2 diabetes, those in whom CD was diagnosed before T1DM, patients with IgA deficiency, and those with incomplete data were excluded.

### 2.3 Data collection

Collected data included age, sex, age at T1DM diagnosis, age at CD diagnosis, annual mean HbA1c levels, notably IgA anti-tTG antibodies, and histological reports of intestinal biopsies.

All information was anonymized and coded before analysis.

### 2.4 Laboratory testing methods

CD screening involved the detection of IgA anti-tissue transglutaminase (anti-tTG) antibodies by enzyme-linked immunosorbent assay (ELISA). Serologically positive cases were confirmed by upper endoscopy with duodenal biopsies, interpreted using the Marsh–Oberhuber classification. A diagnosis of CD was confirmed when Marsh type I to III lesions were present in conjunction with a positive serology.

### 2.5 Statistical analysis

The analyses were performed using IBM SPSS Statistics, versions 22.0 and 27.0. The descriptive statistics were presented as mean ± standard deviation and percentages. Normality was assessed using the Shapiro–Wilk test, which showed non-normal distribution (P < 0.05).

The Mann–Whitney U test was employed to compare distributions between the two groups (CD-absent vs. CD-present).

This retrospective study drew on records from the Department of Pediatrics under the initial authorization of the department head. Parents/guardians provided written informed consent, and all data were fully anonymized and processed under strict confidentiality. Retroactive approval has been obtained from the Ethics Committee of Mustapha Bacha University Hospital Center (reference number: 07/AG/2025).

## 3. RESULTS

### 3.1 Clinical characteristics of the overall cohort

Initially, 387 medical records were reviewed; 278 were excluded because celiac serology results were unavailable. Accordingly, 109 records were included in the analysis.

The review of medical records from 109 patients with T1DM revealed a mean age of 9.99 ± 4.05 years, a mean weight of 36.04 ± 15.23 kg, and a mean height of 136.6 ± 21.4 cm, along with a slight male predominance (53.2%). The mean age at T1DM diagnosis was 6.33 ± 3.45 years, with most cases occurring between 4 and 8 years of age, and a peak incidence at 5 years, suggesting that most diagnoses are made during early childhood. However, cases diagnosed before the age of 2 years were also observed.

**Figure 1.**
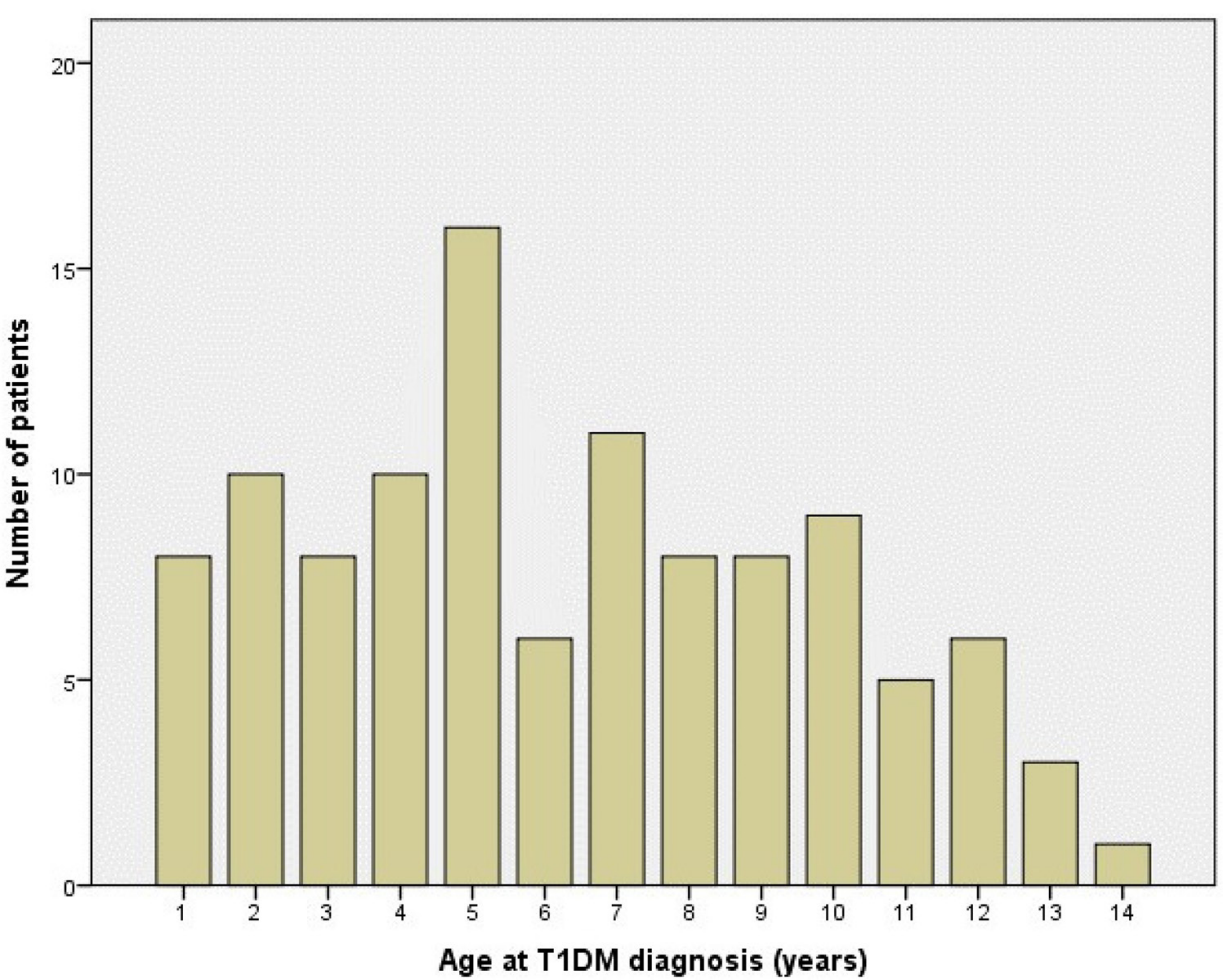
Distribution of age at T1DM diagnosis (years) for the entire cohort; age classes represent 1-year intervals.

However, cases diagnosed before the age of 2 years and up to 14 years were also observed, reflecting a moderate spread around the mean (Figure 1).

### 3.2 T1DM and CD: Diagnosis of CD

**Figure 2.**
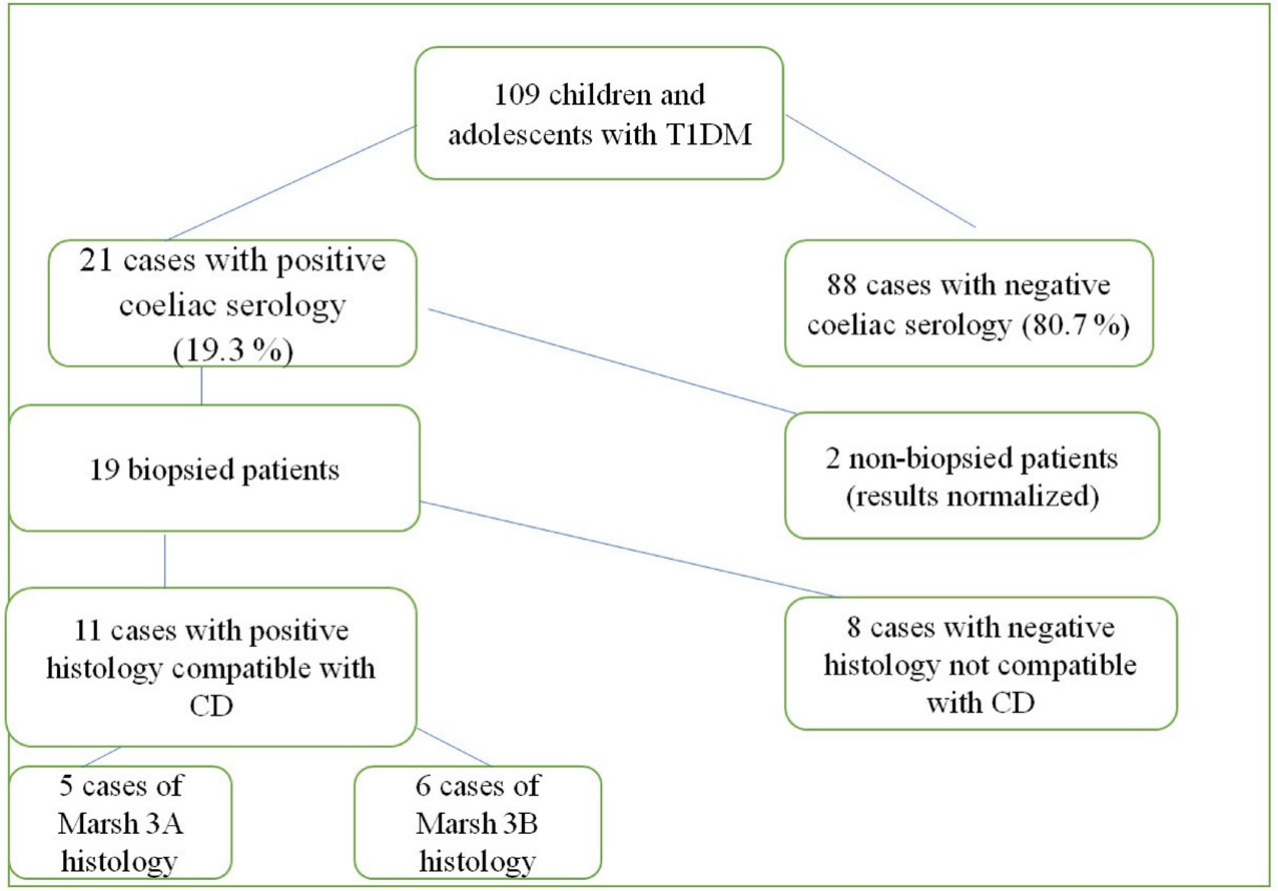
Steps in the diagnosis of celiac disease.

Among the 109 patients with T1DM, 21 subjects had positive serology (19.3%); histological confirmation through biopsy was obtained in 11 cases (57.89%; Figure 2).

### 3.3 T1DM and CD: Comparative analysis and the influence of age at T1DM diagnosis

A comparative analysis was performed between patients with isolated T1DM (*n* = 98) and those with coexisting T1DM and CD (*n* = 11), revealing a significant difference in the age at T1DM diagnosis (P = 0.027). In addition, the mean age at CD diagnosis among patients with coexisting T1DM and CD was 5.82 ± 2.82. However, no significant differences were observed in weight, height, mean age, sex, or the mean of HbA1c (P > 0.05; Table 1).

The logistic regression showed that age at T1DM diagnosis was significantly associated with the risk of CD. For each additional year of age at T1DM diagnosis, the risk of developing CD decreased by 21.4% (odds ratio [OR] = 0.786 [95% CI, 0.627–0.986]; P = 0.038; Table 2).

## 4. DISCUSSION

Our observational study describes the demographic and clinical characteristics of children with T1DM, with or without associated CD, aiming to identify potential factors influencing this co-occurrence. Globally, approximately 1.8 million children and adolescents are living with T1DM.^10^ In Algeria, specifically in the Tlemcen region in the northwest, the incidence is reported at 38.5 per 100,000 children, which is considered extremely high.^11^

The comparison between patients with T1DM only (98 cases) and those with coexisting CD (11 cases) revealed a significant difference in the age at T1DM diagnosis.

The age at T1DM diagnosis among patients who developed CD was 4.18 ± 2.99 years, whereas it was 6.58 ± 3.42 years in children without CD (P = 0.027). The difference between the two groups was statistically significant. These results suggest that children who later develop CD are diagnosed with T1DM at an earlier age compared to those who do not.

These findings support the hypothesis that shared genetic or environmental factors may contribute to the pathogenesis of these diseases in young children.^12^ Multiple studies^13–15^ have reported findings consistent with the observed difference in age at T1DM diagnosis between patients with and without CD. Notably, the study by James et al.^13^ found that children with CD were diagnosed with T1DM at a significantly younger age (7.6 ± 4.4 years) compared to those without CD (9.2 ± 4.4 years; P < 0.001). By contrast, one study reported no such difference.^16^

A longitudinal cohort study of 5295 Swedish children with T1DM reported a biopsy-confirmed prevalence of CD of 9.8%. The highest prevalence was observed among those diagnosed with T1DM before the age of 5 years, reaching 15.0%, supporting the notion that CD is more prevalent in younger children.^17^ Furthermore, two studies involving 478 children also demonstrated a higher prevalence of CD in those diagnosed with type 1 diabetes before the age of 5 years.^18^ Occurrence more than 10 years after T1DM onset appears to be rare.^19^

The analysis of age at T1DM diagnosis revealed a statistically significant association with the presence of CD (OR = 0.786 [95% CI, 0.627–0.986]; P = 0.038).

An OR <1 indicates that younger age at T1DM diagnosis is associated with a higher risk of developing CD.

This inverse relationship may be explained by a more active autoimmune response in early childhood, driven by immune system immaturity and a shared genetic predisposition to both conditions.^12^

Indeed, numerous studies suggest that genetic predisposition may be a key determinant, particularly since both diseases share associations with specific class II HLA alleles, especially HLA-DQ2 and HLA-DQ8.^14,17^

This retrospective study is limited by incomplete data that led to the exclusion of some patients. The small sample size of patients with both T1DM and CD may limit the generalizability of the findings and the statistical detection of small differences. Additionally, the limited number of events can lead to instability in the model’s coefficients, which in turn can cause overestimation or underestimation of the observed effects. Therefore, these findings should be considered preliminary. Future multicenter longitudinal studies with larger cohorts are necessary to confirm these findings and may help clarify the genetic and environmental factors involved in the coexistence of T1DM and CD.

A multidisciplinary approach, combining diabetology, gastroenterology, and dietetics, plays a key role in providing coordinated care and enhancing the quality of life for children with T1DM and CD.

## 5. CONCLUSION

This retrospective study in children and adolescents confirms that CD is a non-negligible comorbidity in T1DM. T1DM and CD share a common autoimmune background, which helps explain their frequent co-occurrence, and our data further show that younger age at T1DM diagnosis is linked to a higher risk of CD. However, it is important to consider these findings as preliminary and require further studies.

Clinically, undiagnosed or untreated CD may contribute to faltering growth, iron-deficiency anemia, reduced bone health, and glycemic instability. In practice, these findings further underscore the benefit of implementing systematic screening for CD at regular intervals in children with T1DM, thereby minimizing the risk of complications from undiagnosed CD. Systematic screening enables early detection and timely, appropriate management, which, together with multidisciplinary care involving diabetology, gastroenterology, and dietetics, ultimately improves overall health outcomes in patients with T1DM.

## ACKNOWLEDGMENTS

The authors would like to thank all the participants and the medical staff of the Pediatric Department at CHU Mustapha Bacha for their valuable contribution to this study.

## FUNDING

This work was not supported by the Fund for Scientific Research.

## CONFLICT OF INTEREST

The authors declare there are no conflicts of interest related to this study.

## ETHICAL APPROVAL

This retrospective study was based on the medical records of patients followed up in the Department of Pediatrics. Parents provided informed consent for the use of the data. All data were anonymized and treated as strictly confidential. Retroactive approval was granted by the ethics committee.

## AUTHOR CONTRIBUTIONS

DAC conducted the research process and took the lead in drafting the manuscript. BMB supervised the study and contributed to the final manuscript review. RB facilitated access to clinical data, provided clinical input, and reviewed the manuscript. MN provided explanations regarding the clinical follow-up of patients.

## DISCLOSURE OF AI USE

The authors used AI tools solely for language refinement and clarity. No AI was used to generate original scientific content or interpret results. All final decisions were made by the authors.

## DATA AVAILABILITY STATEMENT

Due to patient confidentiality and institutional policy, the data sets from this retrospective chart review are not publicly available.

## Figures and Tables

**Table 1.** Comparative analysis of characteristics between patients with T1DM alone and those with T1DM and CD.

	Absence of CD (mean ± SD)	Presence of CD (mean ± SD)	Absence of CD (median (IQR))	Presence of CD (median (IQR))	Mann–Whitney *U* test	*P* value
*n* = 109	98	11	98	11		
Sex	1.46 ± 0.50	1.55 ± 0.52	1 (1)	2 (1)	492.5	0.588
Weight (kg)	36.12 ± 15.43	35.42 ± 13.88	32.60 (23.8)	32 (23.6)	533.5	0.956
Height (cm)	136.53 ± 21.59	137.09 ± 21.11	138 (32)	138 (30)	518	0.833
Age at T1DM diagnosis (years)	6.58 ± 3.42	4.18 ± 2.99	6 (5)	4 (7)	319.5	**0.027**
Age at CD screening (years)	8.62 ± 3.80	5.82 ± 2.82	8 (5)	6 (5)	311.5	**0.022**
Mean HbA1c (%)	8.52 ± 1.43	8.02 ± 1.26	8.27 (1.5)	8 (1)	391.5	0.138

Data are presented as mean ± SD for continuous variables and n (%) for categorical variables. Comparisons were made using the Mann–Whitney *U* test.

T1DM, type 1 diabetes mellitus; CD, celiac disease; IQR, interquartile range.

Bold values indicate statistical significance (P < 0.05).

**Table 2.** Influence of age at T1DM diagnosis on CD onset.

Variable	OR (Exp(B))	95% CI	*P* value (sig.)
Age at diagnosis of T1DM	0.786	(0.627–0.986)	**0.038**

Results of the multivariate logistic regression are presented as OR with 95% confidence intervals (CI) and *P* values.

Bold values indicate statistical significance (*P* < 0.05).

T1DM, type 1 diabetes mellitus; CD, celiac disease; OR, odds ratio.
